# Relative abundance of chemical forms of Cu(II) and Cd(II) on soybean roots as influenced by pH, cations and organic acids

**DOI:** 10.1038/srep36373

**Published:** 2016-11-02

**Authors:** Qin Zhou, Zhao-dong Liu, Yuan Liu, Jun Jiang, Ren-kou Xu

**Affiliations:** 1 State Key Laboratory of Soil and Sustainable Agriculture, Institute of Soil Science, Chinese Academy of Sciences, P.O. Box 821, Nanjing, PR China; 2 University of Chinese Academy of Sciences, Beijing 100049, PR China

## Abstract

Little information is available on chemical forms of heavy metals on integrate plant roots. KNO_3_ (1 M), 0.05M EDTA at pH6 and 0.01 M HCl were used sequentially to extract the exchangeable, complexed and precipitated forms of Cu(II) and Cd(II) from soybean roots and then to investigate chemical form distribution of Cu(II) and Cd(II) on soybean roots. Cu(II) and Cd(II) adsorbed on soybean roots were mainly exchangeable form, followed by complexed form, while their precipitated forms were very low under acidic conditions. Soybean roots had a higher adsorption affinity to Cu(II) than Cd(II), leading to higher toxic of Cu(II) than Cd(II). An increase in solution pH increased negative charge on soybean and thus increased exchangeable Cu(II) and Cd(II) on the roots. Ca^2+^, Mg^2+^ and NH_4_^+^ reduced exchangeable Cu(II) and Cd(II) levels on soybean roots and these cations showed greater effects on Cd(II) than Cu(II) due to greater adsorption affinity of the roots to Cu(II) than Cd(II). L-malic and citric acids decreased exchangeable and complexed Cu(II) on soybean roots. In conclusion, Cu(II) and Cd(II) mainly existed as exchangeable and complexed forms on soybean roots. Ca^2+^ and Mg^2+^ cations and citric and L-malic acids can potentially alleviate Cu(II) and Cd(II) toxicity to plants.

Industrial activities and agricultural practices, such as the application of chemical fertilizers, liming materials, manure, sewage sludge and composts, lead to soil pollution from heavy metals. Accumulation of heavy metals in soils can cause soil degradation and decreased crop yields, and produce long-term risks to ecosystems and human health^1^. However, the mobility and bioavailability of heavy metals are known to be closely related to the processes of adsorption and desorption of metals by and from soil constituents. Extensive investigations have been conducted into the adsorption of heavy metals by soils and soil constituents such as clay minerals^2^,^3^,^4^, iron and aluminum oxides^5^, and organic matter^6^,^7^. Electrostatic adsorption, specific adsorption and surface precipitation were main mechanisms for the adsorption of heavy metals by soils^8^,^9^. Neutral salt such as KNO_3_ was used to extract the heavy metals adsorbed by soils electrostatically through cation exchange reaction^10^. Neutral salt extractants were also used to estimate the phytoavailable trace metals in soils^11^. Meanwhile, EDTA was used to extract the heavy metals adsorbed specifically by soil organic amendments and crop straw biochars^12^,^13^. However, it is difficult to distinguish the specific adsorption of heavy metals from their precipitation in soils quantitatively at present.

Heavy metals in soils reach the root surfaces of plants through the root/soil interface and are then adsorbed and absorbed by plant roots^14^. The root/soil interface therefore presents a barrier to the movement of heavy metals from the soil into plant tissue. There were some investigations involved in the adsorption of heavy metals of Cu(II), Pb(II), Cd(II), Ni(II), Zn(II) and Cr(III) by fine power of plant root materials^15^,^16^,^17^,^18^. In these investigations, plant roots were used as bio-adsorbents to remove the heavy metals from wastewater and natural water. Cation exchange and complex formation with functional groups on plant roots were main mechanisms for biosorption of heavy metals by plant root materials^16^,^17^. There were extensive investigations on the adsorption of heavy metals by cell walls and cell plasma membranes of plant roots as related to rhizotoxicity and uptake of heavy metals by plant roots^19^,^20^,^21^,^22^,^23^,^24^,^25^. It was found that electric potential induced by surface charge on plasma membranes was one of main factors determining ion activities of heavy metals on plasma membranes and their toxicity to plants^20^,^21^,^22^,^23^. However, the investigations on the adsorption of heavy metals by entire plant roots as related to their toxicity and uptake to and by plants were rarely found in literature^26^,^27^. The relative abundance of the various chemical forms of heavy metal cations on plant roots affects the toxicity of metals to plants and the uptake of these metals by plant roots. While, few studies have investigated this topic. The mechanisms by which heavy metals are adsorbed by plant roots and the relative abundance of the chemical forms of adsorbed heavy metals on the roots need to be better understood.

Cu(II) is mainly adsorbed by soils and soil components specifically^28^, while Cd(II) is adsorbed mainly through electrostatic mechanisms and is found in the exchangeable form on soil particles^29^. The different chemical forms of heavy metals exhibit differing bioavailability^30^. Similarly, Nishizono *et al*.^31^ found that the cell walls of plant roots showed a higher affinity to Cu^2+^ than that to Zn^2+^ and Cd^2+^ 31. The cations of the various heavy metals may also be adsorbed by plant roots through different mechanisms. Cu(II) and Cd(II) were selected for this study to examine the relative abundance of their various chemical forms on soybean roots after adsorption.

Carboxyl and phenolic groups are commonly found on plant roots^32^. The deprotonation of these acidic groups gives plant roots a negative charge and enables roots to adsorb heavy metals through electrostatic interaction. In addition, these acidic groups have a strong complexing ability and thus can adsorb heavy metals specifically through the formation of surface complexes with metal cations on the plant root surfaces^33^. The degree of dissociation of weak acids increases with increasing system pH, so that pH not only affects the charge characteristics of a root surface, but can also affect the hydrolysis of heavy metal cations in solution, thereby affecting their reaction with functional groups on the root surface.

Environmental stress causes plant roots to actively or passively release soluble organic acids into the rhizosphere. Organic acids contain a large quantity of carboxyl functional groups and form soluble complexes with heavy metal cations^34^. Consequently, organic acids may affect adsorption and combinations of heavy metals form on the root surface. Exogenous cations of Ca^2+^, Mg^2+^ and NH_4_^+^ effectively inhibit absorption of Mn(II) by barley and thus reduce Mn(II) toxicity to the plant^35^. Cations such as Ca^2+^, Mg^2+^ and NH_4_^+^ compete with Al^3+^ for adsorption sites and inhibit adsorption of Al^3+^ by the roots of soybean and maize^33^. Exogenous cations may also inhibit the adsorption of heavy metal cations by plant roots and affect the relative abundance of the chemical forms of heavy metals on plant roots. However, the effects of cations and organic acids on the relative abundance of the chemical forms of heavy metals on plant roots requires further research.

The objectives of this study were: (1) to investigate the relative abundance of the various chemical forms of Cu(II) and Cd(II) on soybean roots after adsorption by the roots; and (2) to examine the effects of exogenous cations and organic acids on the relative abundance of the chemical forms of Cu(II) and Cd(II) on soybean roots. The findings should improve our understanding of the chemical behaviors of these heavy metals at the root/soil interface, and suggest means of reducing the toxicity of heavy metals to plants.

Neutral salts were previously used to extract exchangeable heavy metals adsorbed on soils and exchangeable Al from plant roots^10^,^33^. EDTA was used to extract heavy metal cations adsorbed specifically by soil organic matter and crop straw biochars in previous studies^12^,^13^. The mechanism for specific adsorption of Cu(II) and Cd(II) on soybean roots was formation of surface complexes of the metals with functional groups on the roots, similar to the specific adsorption of heavy metals by organic matter and biochars. HCl was used to extract the precipitates of Al hydroxides from plant roots in previous studies^33^,^36^. Cu(II) and Cd(II) mainly formed the precipitates of hydroxides on soybean roots in present study. Therefore, the neutral salt of KNO_3_, EDTA, and HCl were used sequentially to extract the exchangeable, complexed and precipitated forms of Cu(II) and Cd(II) from soybean roots in this study.

## Results and Discussion

### The relative abundance of the chemical forms of Cu(II) and Cd(II) on soybean roots

**Figure 1:**
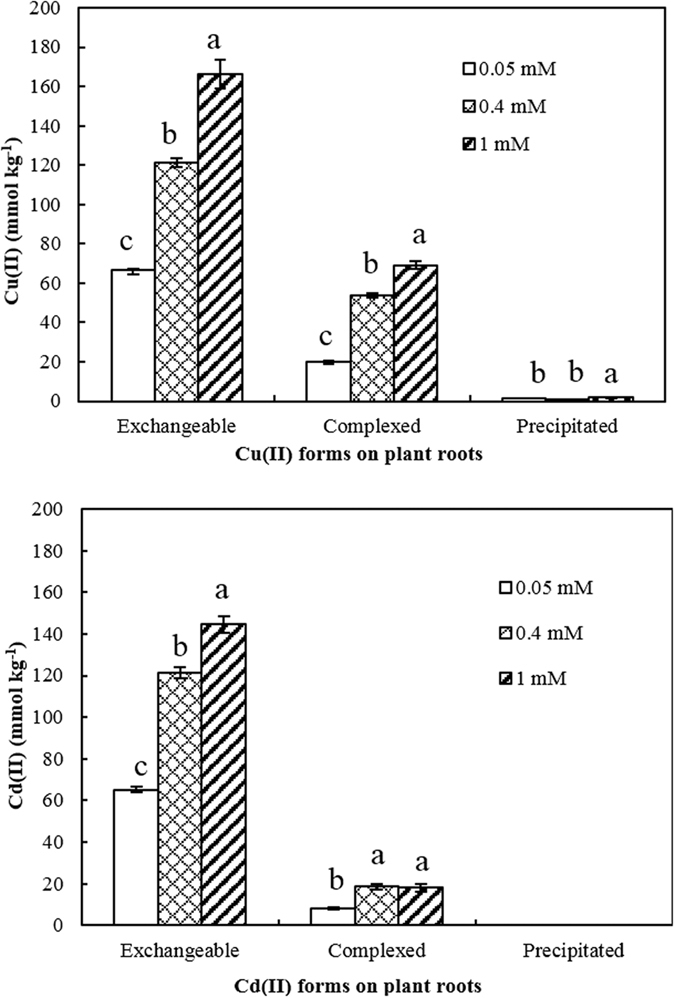
Effects of the initial concentration of Cu(II) and Cd(II) on their adsorption and chemical forms on 14-day-old roots of soybean at pH 4.2.

Cu(II) and Cd(II) adsorbed onto soybean roots could be differentiated into exchangeable, complexed, and precipitated forms. Under strong acidic conditions of pH 4.2, Cu(II) and Cd(II) was mainly found on soybean roots in the exchangeable forms, followed by complexed forms, while the amounts of precipitated Cu(II) and Cd(II) were very low (Fig. 1). For example, when the initial concentration of Cu(II) and Cd(II) was 0.4 mM, the exchangeable Cu(II) and Cd(II) accounted for 68.9% and 86.8% of total adsorbed Cu(II) and Cd(II), and the complexed Cu(II) and Cd(II) accounted for 30.5% and 13.2% of total adsorbed Cu(II) and Cd(II), respectively. Both the exchangeable and complexed Cu(II) increased significantly with increasing initial Cu(II) concentration (*P* < 0.05). Similarly, the exchangeable Cd(II) was significantly increased (*P* < 0.05) as the initial concentration of Cd(II) rose. The complexed Cd(II) increased significantly as its initial concentration increased from 0.05 mM to 0.4 mM (*P* < 0.05), while the increase was not significant when the initial concentration increased from 0.4 mM to 1.0 mM. Comparing the two heavy metals, the amount of exchangeable Cu(II) was almost the same as that of the exchangeable Cd(II) at initial concentrations of 0.05 and 0.4 mM. However, the amount of complexed Cu(II) was much higher than that of complexed Cd(II) (Fig. 1). For example, at initial concentrations of 0.05, 0.4 and 1.0 mM, the amount of exchangeable Cu was 1.02, 0.99, and 1.15 times the content of exchangeable Cd(II) respectively, while the content of complexed Cu(II) was 2.43, 2.89, 3.82 times the content of complexed Cd(II), respectively. These results suggest that the adsorption affinity of soybean roots to Cu(II) was much greater than to Cd(II), leading to the higher specific adsorption capacity of the roots to Cu(II) than to Cd(II). This resembles the Cu(II) and Cd(II) adsorption capacity of soil organic matter and crop straw biochars^13^,^37^, caused by the stronger complexing ability of organic functional groups with Cu(II) than with Cd(II)^38^.

**Figure 2:**
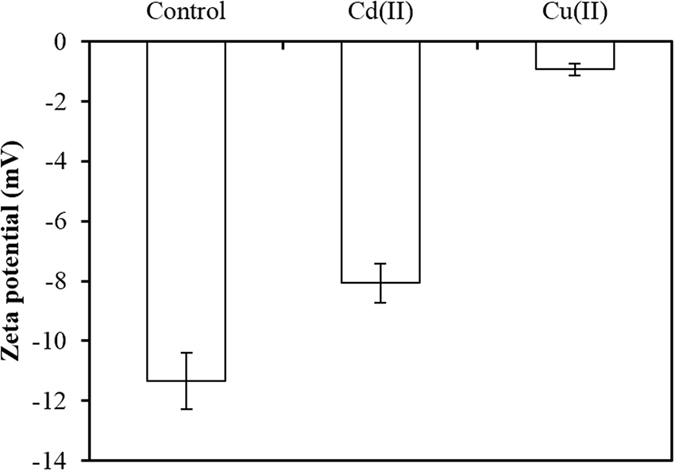
Zeta potential of 14-day-old soybean roots with and without adsorbed Cu(II) and Cd(II). Zeta potential was measured using the streaming potential method at pH 4.2 and NaCl was used as the electrolyte solution.

The effect of Cu(II) and Cd(II) on the zeta potential of soybean roots provides evidence for the differences between the formation of surface complexes of Cu(II) and Cd(II) with organic functional groups on soybean roots. The zeta potential of soybean roots was negative (Fig. 2), suggesting that soybean roots carry a negative charge. The adsorption of Cu(II) and Cd(II) by soybean roots reduced the negativity of the zeta potential, suggesting that surface complexes of Cu^2+^ and Cd^2+^ with functional groups form on the root surfaces. This resembles the effect of the formation of surface complexes of Cu^2+^ with surface functional groups on the zeta potential observed on crop straw biochars^39^. The adsorption of Cu(II) led to a greater change in zeta potential of soybean roots than did the adsorption of Cd(II) (Fig. 2), suggesting that a greater number of Cu(II) complexes formed than those of Cd(II) on soybean roots. This was consistent with the relative abundance of complexed Cu(II) and Cd(II) on soybean roots (Fig. 1).

**Figure 3:**
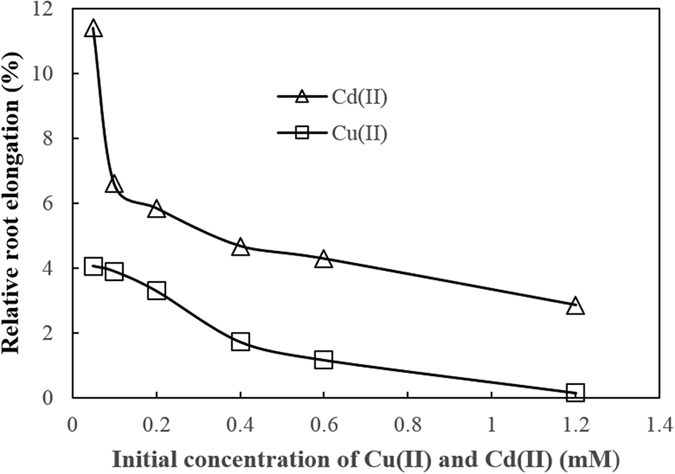
Effects of Cu(II) and Cd(II) on the elongation of soybean roots in solution culture conditions.

The abundance of the chemical forms of Cu(II) and Cd(II) on soybean roots may be related to their toxicities to the plant. The effects of different concentrations of Cu(II) and Cd(II) on soybean root elongation were compared and the results are shown in Fig. 3. Root elongation was significantly inhibited with the increasing concentrations of these heavy metals, suggesting that increasing amounts of exchangeable and complexed Cu(II) and Cd(II) on soybean roots increased their toxicities to the plant and the resulting inhibition of soybean root growth. The inhibiting effect of Cu(II) on root elongation was greater than that of Cd(II) at the same concentration, suggesting that Cu(II) was more toxic to the plant than Cd(II). This was consistent with previous observations by other researchers^40^. The amount of exchangeable Cu(II) on soybean roots was almost the same as exchangeable Cd(II), but complexed Cu(II) was much more abundant than complexed Cd(II), which may account for the greater toxicity of Cu(II) to soybean than Cd(II).

### Effect of pH on the relative abundance of the chemical forms of Cu(II) and Cd(II)

**Figure 4:**
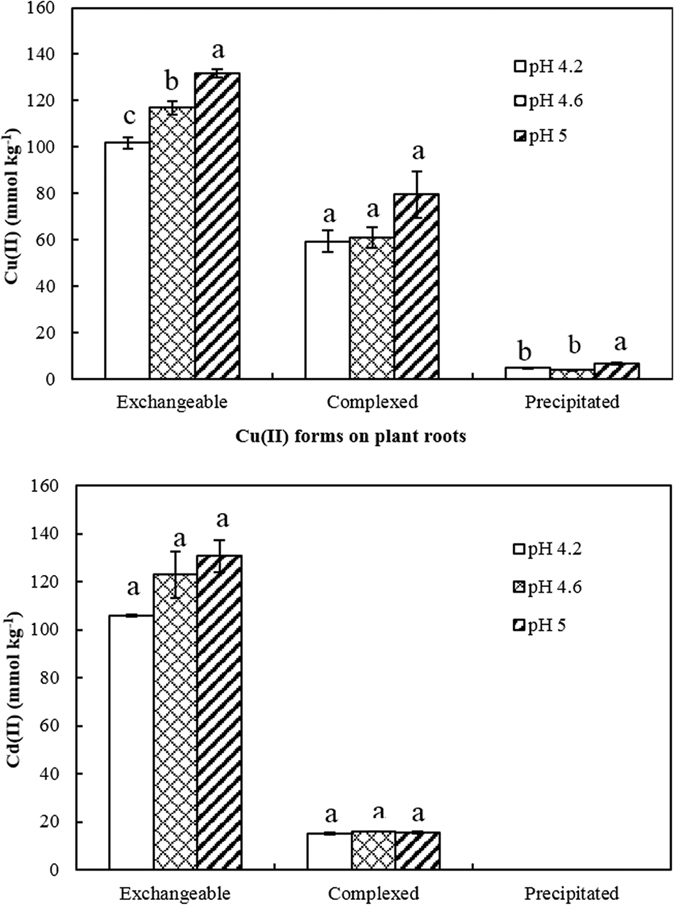
Effect of solution pH on the adsorption of Cu(II) and Cd(II) and their chemical forms on 14-day-old roots of soybean (Initial concentration of Cu(II) and Cd(II) was 0.4 mM).

As the pH increased, the amount of exchangeable Cu(II) on soybean roots increased significantly (*P* < 0.05) (Fig. 4), suggesting that the increased negative charge on the roots induced by increasing pH led to an increase in exchangeable Cu(II). The amount of complexed Cu(II) also increased with increasing pH, but the increase was not significant (Fig. 4). Increasing pH enhanced dissociation of acidic functional groups on the roots and hence the complexing ability of the functional groups with Cu(II). The amount of exchangeable Cd(II) also increased with increasing pH, although not to a significant degree. The increase in pH did not affect the complexed Cd(II) on the roots. The effect of pH on Cu(II) adsorption by soybean roots was greater than on Cd(II) adsorption, suggesting that Cu(II) adsorption was more sensitive to pH change than Cd(II) adsorption.

**Figure 5:**
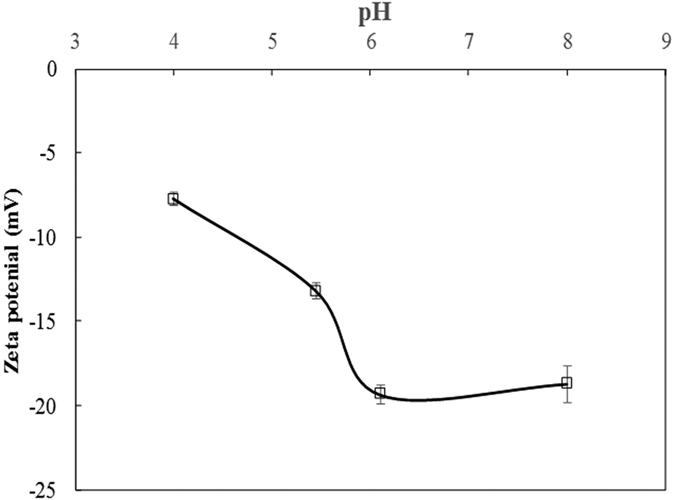
Zeta potential of 14-day-old soybean roots measured using the streaming potential method at various pH levels.

Figure 5 indicates that the deprotonation of the acidic functional groups on soybean roots increased with increasing pH, increasing the negative charge on the roots and resulting in increased negative zeta potential of the roots. This provides evidence for the increases of exchangeable Cu(II) and Cd(II) on soybean roots with increasing pH, as shown in Fig. 4.

Figure 4 also shows a greater amount of precipitated Cu(II) on soybean roots than precipitated Cd(II), due to the greater hydrolyzing ability of Cu^2+^ compared with Cd^2+^. The hydrolysis constant (pK) is an important parameter in characterizing the hydrolyzing ability of heavy metal cations. Smaller pK values indicate stronger hydrolyzing potential. The pK values of Cu^2+^ and Cd^2+^ reported in the literature are 6.5 and 9.7, respectively^41^, indicating that the hydrolyzing ability of Cu^2+^ is greater than Cd^2+^. This accounts for the greater amount of precipitated Cu(II) at pH 5 than at pH 4.2 and 4.6.

### Cation effects on the relative abundance of the chemical forms of Cu(II) and Cd(II)

**Figure 6:**
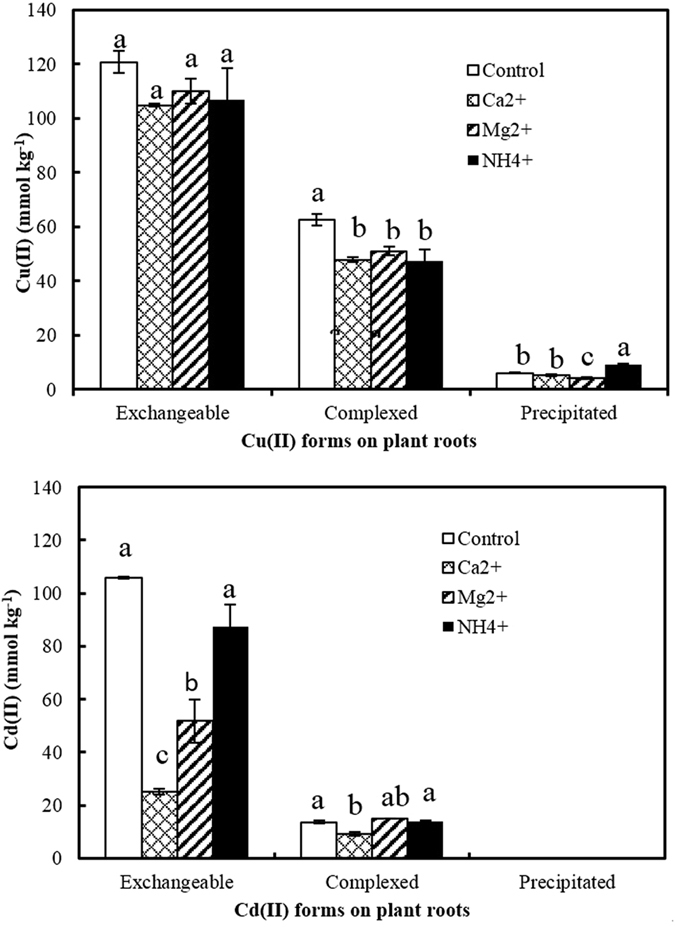
Effects of cations on adsorption of Cu(II) and Cd(II) and their chemical forms on 14-day-old roots of soybean at pH 4.2.

The addition of exogenous Ca^2+^, Mg^2+^ and NH_4_^+^ significantly reduced the exchangeable Cu(II) on soybean roots by 13.2%, 8.8% and 11.0 %, respectively, compared with the control (Fig. 6), but the decrease in exchangeable Cu(II) was not statistically significant. Ca^2+^ and Mg^2+^ significantly reduced the exchangeable Cd(II) on soybean roots by 76.1% and 51.1% (*P* < 0.05). NH_4_^+^ decreased the exchangeable Cd(II) by 17.7% compared with the control, but this decrease was not significant. Cations of Ca^2+^, Mg^2+^ and NH_4_^+^ decreased exchangeable Cu(II) and Cd(II) on soybean roots through competition with Cu(II) and Cd(II) for exchangeable sites on negatively charged roots. The presence of these cations led to a greater decrease in exchangeable Cd(II) than exchangeable Cu(II) due to the greater adsorption affinity of the roots to Cu(II) than Cd(II), as mentioned above. Of the three cations, Ca^2+^ and Mg^2+^ had a much greater effect on exchangeable Cd(II) than did NH_4_^+^ due to the higher competitive ability for adsorption sites of the bivalent cations of Ca^2+^ and Mg^2+^ compared with the monovalent cation NH_4_^+^. Ca^2+^, Mg^2+^ and NH_4_^+^ significantly reduced the amount of complexed Cu(II) by 23.5%, 18.4% and 24.3%, respectively, compared with the control (*P* < 0.05). Ca^2+^ significantly reduced the amount of complexed Cd(II), while Mg^2+^ and NH_4_^+^ had no significant effect compared with the control (Fig. 6).

The presence of cations of Ca^2+^, Mg^2+^ and NH_4_^+^ inhibited the adsorption of Cu(II) and Cd(II) by soybean roots and may therefore decrease the toxicities of these metals to the plant and their absorption by soybean roots. The observations of Hardiman *et al*.^42^ support this conclusion^42^. They found that the presence of base cations inhibited the uptake of Cd(II) by soybean roots and the effects of these cations were ranked: Ca^2+^ > Mg^2+^ > K^+^ > Na^+^.

### Effects of organic acids on the relative abundance of the chemical forms of Cu(II) and Cd(II)

**Figure 7:**
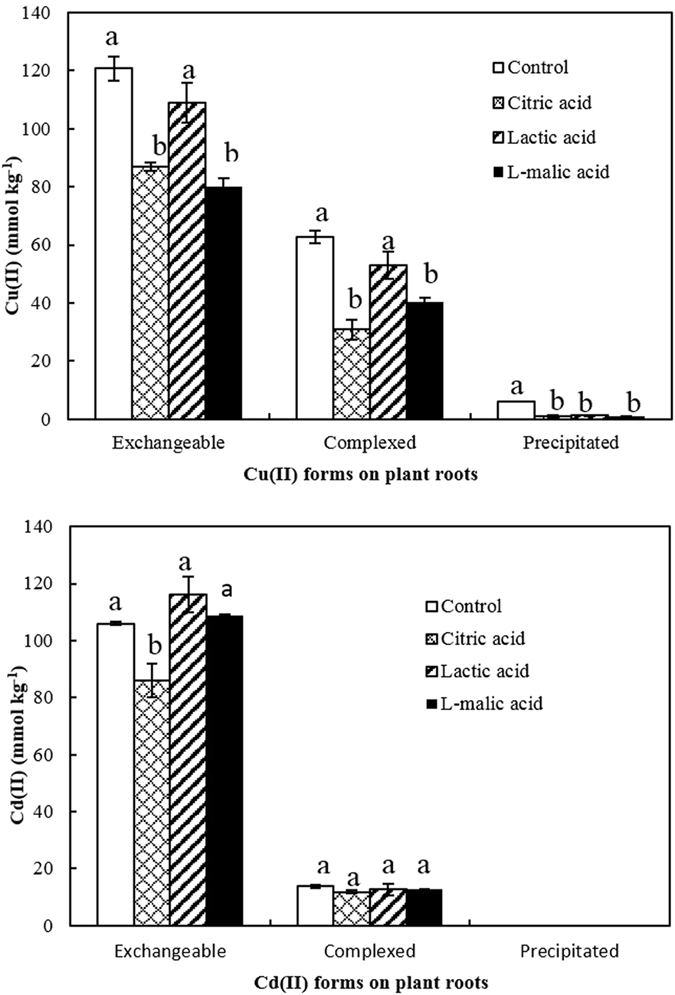
Effects of organic acids on adsorption of Cu(II) and Cd(II) and their chemical forms on 14-day-old roots of soybean at pH 4.2.

The effects of low-molecular-weight (LMW) organic acids on the relative abundance of the chemical forms of Cu(II) and Cd(II) are shown in Fig. 7. The presence of L-malic and citric acids significantly decreased the amounts of exchangeable and complexed Cu(II) on soybean roots compared with the control (*P* < 0.05). Lactic acid also reduced the amounts of exchangeable and complexed Cu(II) on soybean roots, but the decrease in exchangeable and complexed Cu(II) was not significant. The three organic acids also decreased the amount of precipitated Cu(II) on soybean roots (Fig. 7). During adsorption experiments of Cu(II) and Cd(II), the solution pH was kept constant at 4.2. Therefore, the effects of these organic acids on the relative abundance of the chemical forms of Cu(II) and Cd(II) on soybean roots were mainly attributed to their complexation with both metal cations in solutions. The stability constants (logK) for complexes of citric, L-malic and lactic acids with Cu^2+^ are 5.9, 3.33 and 3.02, respectively^43^,^44^. Compared with lactic acid, the stronger complexing ability of citric and L-malic acids led to higher levels of Cu(II) remaining in the nutrient solution through the formation of more complexes with Cu(II) and thus increased their inhibitions on Cu(II) adsorption by soybean roots. The formation of complexes between the organic acids and Cu^2+^ also inhibited the precipitation of Cu(II) on soybean roots (Fig. 7).

The presence of citric acid significantly decreased the amount of exchangeable Cd(II) (*P* < 0.05), but did not reduce the amount of complexed Cd(II) significantly, compared with the control (Fig. 7). Both L-malic and lactic acids did not significantly decrease the amounts of exchangeable and complexed Cd(II) on soybean roots. These organic acids showed less effect on the adsorption and relative abundance of the chemical forms of Cd(II) compared with Cu(II), due to their relative lower complexing ability with Cd(II) than with Cu(II). The stability constants (logK) for complexes of citric, L-malic and lactic acids with Cd^2+^ are 3.15, 1.34 and 1.07, respectively^43^,^45^, much lower than these for the complexes with Cu^2+^. The logK of citric acid with Cd^2+^ is greater than those of L-malic and lactic acids with Cd^2+^, and is responsible for the significant decrease in exchangeable Cd(II) caused by citric acid. LMW organic acids arise mainly from the exudates of plant roots and the various stages of decomposition of plant residues in soils^34^. These organic acids can form complexes with heavy metal cations in soil solutions and thus inhibit the adsorption of heavy metals onto plant roots, which may also decrease the availability and toxic effect of these metals on plants, especially in the case of Cu(II) due to the greater logK of organic acid complexes with Cu^2+^.

## Conclusions

Cu(II) and Cd(II) adsorbed on soybean roots mainly occurred in the exchangeable forms, followed by complexed forms, while the precipitated forms occurred at low levels under acidic conditions. Soybean roots had a higher adsorption affinity to Cu(II) than Cd(II), leading to greater toxicity of Cu(II) to the plant than Cd(II). The formation of complexes of Cu^2+^ and Cd^2+^ with functional groups on soybean roots reduced the negative level of the zeta potentials and the adsorption of Cu(II) showed a greater effect on the zeta potential of soybean roots than did the adsorption of Cd(II). The increase in solution pH increased the negative charge on soybean roots and thus increased the amounts of exchangeable Cu(II) and Cd(II) on the roots. Cu(II) adsorption by soybean roots was more affected by pH than was Cd(II) adsorption. The presence of Ca^2+^, Mg^2+^ and NH_4_^+^ decreased the amounts of exchangeable Cu(II) and Cd(II) on soybean roots through competition for exchangeable sites on negatively charged roots with Cu^2+^ and Cd^2+^ and the cations showed greater effects on Cd(II) than Cu(II) due to the greater adsorption affinity of the roots to Cu(II) than Cd(II). Ca^2+^ showed the greatest effect on exchangeable Cd(II), followed by Mg^2+^, while NH_4_^+^ showed the least effect on exchangeable Cd(II). The presence of L-malic and citric acid decreased the amounts of exchangeable and complexed Cu(II) on soybean roots. Citric acid decreased the amount of exchangeable Cd(II) on soybean roots. In summary, the cations Ca^2+^ and Mg^2+^ and the LMW organic acids of citric and L-malic acids can decrease the availability of the heavy metals Cu(II) and Cd(II) and potentially alleviate their toxicity to plants.

The differences in adsorption affinity of soybean roots to Cu(II) and Cd(II) led to the differences in the relative abundance of the chemical forms of both metals and thus affected their toxicity to the plant. The exogenous cations of Ca^2+^, Mg^2+^ and NH_4_^+^ and organic acids of citric, L-malic and lactic acids decreased the exchangeable and complexed forms of Cu(II) and Cd(II) on soybean roots to various extents, mainly depending on the competing ability of these cations for adsorption sites with the heavy metals and the complexing ability of these organic acids with Cu^2+^ and Cd^2+^. These findings are of fundamental significance in understanding the chemical behaviors of heavy metals at the root/soil interface, and suggest means of alleviating the toxicity of heavy metals to plants.

## Materials and Methods

### Plant materials and cultivation

Soybean (*Glycine max cv*. Xudou 14) was used in this study. The soybean seeds were surface-sterilized with 10% H_2_O_2_ for 10 min, washed with deionized water three times, soaked for 4 h in deionized water, and then germinated in sterilized sand in the dark. From the fourth day, the plant seedlings were cultivated in a nutrient solution (initial pH 4.2) and grown in a controlled environment growth chamber at a temperature of 27 °C, a day-length of 14 h, a light intensity of 375 μmol photonm^−2^ s^−1^, and a relative humidity of 70%. The composition of the nutrient solution was as follows: (mM) 2.0 (NH_4_)_2_SO_4_, 12.0 NaNO_3_, 1.0 NaH_2_PO_4_·2H_2_O, 3.0 K_2_SO_4_·5H_2_O, 2.0 MgSO_4_·2H_2_O, 4.0 CaCl_2_; (μM) 9.15 MnCl_2_·4H_2_O, 0.32 CuSO_4_·5H_2_O, 0.77 ZnSO_4_·7H_2_O, 0.02 (NH_4_)_6_Mo_7_O_24_·4H_2_O, 46.26 H_3_BO_3_ and 20.0 FeSO_4_·7H_2_O-EDTA. The nutrient solution was changed every two days. Fourteen-day-old roots were used for the Cu(II) and Cd(II) adsorption experiments.

### Cu(II) and Cd(II) adsorption/desorption experiments with entire roots

Before the adsorption experiments, the plant roots were placed in deionized water for 1 h to remove excess nutrient ions from the root surfaces. The plant roots were then separated from the stems, washed with deionized water three times, and then carefully blotted dry. About 8 g of fresh roots were placed in a 300-mesh nylon bag (preliminary experiments showed that the nylon bag did not adsorb Cu(II) and Cd(II)), then immersed in 1-L Cu(II) and Cd(II) solutions and magnetically stirred. After 2 h, the bag containing the plant roots was removed from the adsorption solution and washed with distilled water three times, and any excess water was removed with filter paper. Then the roots were sequentially placed in 1 L 1 M KNO_3_, 0.05 M EDTA at pH 6, and 0.01 M HCl for 1 h each to extract the exchangeable, complexed, and precipitated Cu(II) and Cd(II) from the roots. After each extraction, the roots were washed with distilled water three times and the excess water was blotted off in preparation for the next extraction.

The exchangeable heavy metals were absorbed by the soybean roots by electrostatic attraction and can be exchanged with cations of indifferent electrolytes. High concentration KCl has previously been used to extract exchangeable heavy metals from soils^46^ and from plant roots. The complexed heavy metals comprised the binding state of the metals through the formation of surface complexes with functional groups on the plant roots. The precipitated heavy metals bound to the roots as hydroxyl polymers or oxides formed on the root surfaces. The concentrations of Cu(II) and Cd(II) in the extractants were determined by means of atomic adsorption spectroscopy.

To investigate the effect of the initial concentrations of Cu(II) and Cd(II) on their adsorption and the relative abundance of their chemical forms, 0.05, 0.4 and 1.0 mM of Cu(II) and Cd(II) solutions were used. Similar experimental procedures were then conducted. The solution was kept at pH 4.2 throughout the experiment.

To investigate the effect of pH on the adsorption of Cu(II) and Cd(II) and the relative abundance of their chemical forms, the solution was kept at pH 4.2, 4.6 and 5.0. The initial concentration of Cu(II) and Cd(II) was 0.4 mM. Similar experimental procedures were then conducted.

To investigate the effects of cations on the adsorption of Cu(II) and Cd(II) and the relative abundance of their chemical forms, we prepared solutions containing mixtures of 0.4 mM of Cu(II) or Cd(II) and 1 mM each of Ca^2+^ (CaCl_2_), Mg^2+^ (MgCl_2_) or NH_4_^+^(NH_4_Cl) and carried out similar experimental procedures. The solution pH was kept at 4.2 throughout the experiment.

The effect of organic acid on the adsorption of Cu(II) and Cd(II) and the relative abundance of their chemical forms was investigated by preparing mixed solutions containing 0.4 mM of Cu(II) or Cd(II) and 1 mM each of L-malic acid, lactic acid or citric acid and carrying out similar experimental procedures. The solution pH was kept at 4.2 throughout the experiment.

### The determination of the zeta potential of the root surface

About 10 g of fresh roots were reacted with 0.4 mM solutions of Cu(II) and Cd(II) for 2 h, at a constant solution pH of 4.2. After adsorption of Cu(II) and Cd(II), the roots were washed with distilled water three times and then air-dried. A streaming potential apparatus was used to measure the zeta potential of soybean roots with and without adsorbed Cu(II) and Cd(II)^47^. The air-dried soybean roots were cut to a length of 2 cm, and 0.25 g of the roots were horizontally loaded into a sample cell. NaCl (1.5 L, electrolyte solution) with a conductivity of 80 μS cm^−1^ and pH 4.2 was put into an electrolyte container and then flowed through the soybean roots for 40 min using a magnetic pump as a driver. The used electrolyte solution was then discarded, another 1.5 L of the electrolyte solution was put into the electrolyte container and flowed through the soybean roots for 10 min until equilibrium was reached, after which the flow rate of electrolyte was increased stepwise by adjusting the valve and the streaming potential ΔE and pressure difference ΔP were recorded. The zeta potential of the soybean roots was calculated using the Helmholtz–Smoluchowski equation.

### Root elongation measurement

The roots of 4-day-old seedlings were exposed for 48 h to 0.5 mM CaCl_2_ solutions with 0, 10, 20, 40, 100, 200, 1000 μM of Cu(NO_3_)_2_ and Cd(NO_3_)_2_ at pH 4.2. There were 10 roots in each treatment and their lengths were measured before and after exposure to Cu(II) and Cd(II), and the average lengths were recorded to calculate the relative root elongation (RRE):


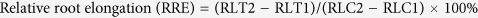


Where RLC1 is the initial root length of untreated controls; RLC2 is the final root length of untreated controls after 48 h (control); RLT1 is the initial root length before exposure; RLT2 is the final root length after exposure to Cu(II) and Cd(II) for 48 h.

### Statistical analysis

SPSS 20.0 was used for statistical analysis and data processing. A one-way analysis of variance was undertaken for all experiments to identify significant differences between treatments.

## Additional Information

**How to cite this article**: Zhou, Q. *et al*. Relative abundance of chemical forms of Cu(II) and Cd(II) on soybean roots as influenced by pH, cations and organic acids. *Sci. Rep.* **6**, 36373; doi: 10.1038/srep36373 (2016).

**Publisher’s note:** Springer Nature remains neutral with regard to jurisdictional claims in published maps and institutional affiliations.

## References

[b1] Chary N., Kamala C. & Raj D. Assessing risk of heavy metals from consuming food grown on sewage irrigated soils and food chain transfer. Ecotox. Environ. Safe. 69, 513–524 (2008). 10.1016/j.ecoenv.2007.04.013 17555815

[b2] Spark K. M., Wells J. D. & Johnson B. B. Characterizing trace metal adsorption on kaolinite. Eur. J. Soil Sci. 46, 633–640 (1995).

[b3] Al-Qunaibit M. H., Mekhemer W. K. & Zaghloul A. A. The adsorption of Cu(II) ions on bentonite–a kinetic study. J. Colloid Interface Sci. 283, 316–321 (2005). 15721900 10.1016/j.jcis.2004.09.022

[b4] Wang S., Dong Y., He M., Chen L. & Yu X. J. Characterization of GMZ bentonite and its application in the adsorption of Pb(II) from aqueous solutions. Appl. Clay Sci. 43, 164–171 (2009).

[b5] Stahl R. S. & James B. R. Zinc sorption by manganese-oxide-coated sand as a function of pH. Soil Sci. Soc. Am. J. 55, 1291–1294 (1991).

[b6] Covelo E. F., Vega F. A. & Andrade M. L. Competitive sorption and desorption of heavy metals by individual soil components. J. Hazard. Mater. 140, 308–315 (2007). 17049729 10.1016/j.jhazmat.2006.09.018

[b7] Gerritse R. G. & Vanel W. The relationship between adsorption of trace metals, organic matter, and pH in temperate soils. J. Environ. Qual. 13, 197–204 (1984).

[b8] Xu R. K. Interaction between heavy metals and variable charge surfaces In Molecular environmental soil science (eds Xu J. M. & Sparks D. L.) 193–228 (Springer, 2013).

[b9] Sparks D. L. Advances in coupling of kinetics and molecular scale tools to shed light on soil biogeochemical processes. Plant Soil 387, 1–19 (2015)

[b10] Xu R. K., Xiao S. C., Zhao A. Z. & Ji G. L. Effect of Cr(VI) on adsorption and desorption behavior of Cu(II) in the colloidal systems of two authentic variable charge soils. J. Colloid Interface Sci. 284, 22–29 (2005). 15752780 10.1016/j.jcis.2004.09.053

[b11] Menzies N. W., Nonn M. J. & Kopittke P. M. Evaluation of extractants for estimation of the phytoavailable trace metals in soils. Environ. Pollut. 145, 121–130 (2007) 16777287 10.1016/j.envpol.2006.03.021

[b12] Santos S. . Influence of different organic amendments on the potential availability of metals from soil: A study on metal fractionation and extraction kinetics by EDTA. Chemosphere 78, 389–396 (2010) 19962175 10.1016/j.chemosphere.2009.11.008

[b13] Xu R. K. & Zhao A. Z. Effect of biochars on adsorption of Cu(II), Pb(II) and Cd(II) by three variable charge soils from southern China. Environ. Sci. Pollut. Res. 20, 8491–8501 (2013). 10.1007/s11356-013-1769-8 23649601

[b14] Toppi L. S. D. & Gabbrielli R. Response to cadmium in higher plants. Environ. Exp. Bot. 41, 105–130 (1999).

[b15] Araú G. C. L., Lemos S. G., Ferreira A. G., Freitas H. & Nogueira A. R. A. Effect of pre-treatment and supporting media on Ni(II), Cu(II), Al(III) and Fe(III) sorption by plant root material. Chemosphere 68, 537–545 (2007). 17280702 10.1016/j.chemosphere.2006.12.054

[b16] Zheng J. C. . Removal of Cu(II) in aqueous media by biosorption using water hyacinth roots as a biosorbent material. J. Hazard. Mater. 171, 780–785 (2009). 19596517 10.1016/j.jhazmat.2009.06.078

[b17] Li X. S., Liu S. L., Na Z. Y., Lu D. N. & Liu Z. Adsorption, concentration, and recovery of aqueous heavy metal ions with the root powder of *Eichhornia crassipes*. Ecol. Eng. 60, 160–166 (2013).

[b18] Jorgetto A. O. . Cassava root husks powder as green adsorbent for the removal of Cu(II) from natural river water. Appl. Surf. Sci. 288, 356–362 (2014).

[b19] Vulkan R., Yermiyahu U., Mingelgrin U., Rytwo G. & Kinraide T. B. Sorption of copper and zinc to the plasma membrane of wheat root. J. Membrane Biol. 202, 97–104 (2004). 15702373 10.1007/s00232-004-0722-7

[b20] Kopittke P. M., McKenna B. A., Blamey F. P. C., Wehr J. B. & Menzies N. W. Metal-induced cell rupture in elongating roots is associated with metal ion binding strengths. Plant Soil 322, 303–315 (2009).

[b21] Wang P., Zhou D. M., Peijnenburg W. J. G. M., Li L. Z. & Weng N. Y. Evaluating mechanisms for plant-ion (Ca^2+^, Cu^2+^, Cd^2+^ or Ni^2+^) interactions and their effectiveness on rhizotoxicity. Plant Soil 334, 277–288 (2010).

[b22] Kopittke P. M., Blamey F. P. C., McKenna B. A., Wang P. & Menzies N. W. Toxicity of metals to roots of cowpea in relation to their binding strength. Environ. Toxicol. Chem. 30, 1827–1833 (2011). 21538487 10.1002/etc.557

[b23] Wang Y. M., Kinraide T. B., Wang P., Zhou D. M. & Hao X. Z. Modeling rhizotoxicity and uptake of Zn and Co singly and in binary mixture in wheat in terms of the cell membrane surface electrical potential. Environ. Sci. Technol. 47, 2831–2838 (2013). 23405885 10.1021/es3022107

[b24] Guigues S. . Isolated cell walls exhibit cation binding properties distinct from those of plant roots. Plant Soil 381, 367–379 (2014).

[b25] Keller C. . Effect of silicon on wheat seedlings (*Triticum turgidum* L.) grown in hydroponics and exposed to 0 to 30 μM Cu. Planta 241, 847–860 (2015). 25515193 10.1007/s00425-014-2220-1

[b26] Kalis E. J. J., Temminghoff E. J. M., Town R. M., Unsworth E. R. & van Riemsdijk W. H. Relationship between metal speciation in soil solution and metal adsorption at the root surface of ryegrass. J. Environ. Qual. 37, 2221–2231 (2008). 18948475 10.2134/jeq2007.0543

[b27] Duffner A., Hoffland E., Weng L. P. & van der Zee S. E. A. T. M. Predicting zinc bioavailability to wheat improves by integrating pH dependent nonlinear root surface adsorption. Plant Soil 373, 919–930 (2013).

[b28] Mclaren R. G. & Crawford D. V. Studies on soil copper. J. Soil Sci. 25, 443–452 (1974).

[b29] Hickey M. G. & Kittrick J. A. Chemical partitioning of cadmium, copper, nickel and zinc in soils and sediments containing high levels of heavy metals. J. Environ. Qual. 13, 372–376 (1984).

[b30] Krishnamurti G. S. R., Huang P. M., van Rees K. C. J., Kozak L. M. & Rostad H. P. M. A new soil test method for the determination of plant-available cadmium in soils. Commun. Soil Sci. Plant Anal. 26, 2857–2867 (1995).

[b31] Nishizono H., Ichikawa H., Suziki S. & Ishii F. The role of the root cell wall in the heavy metal tolerance of Athyrium-yokoscense. Plant Soil 101, 15–20 (1987).

[b32] Meychik N. R., Nikolaeva J. I. & Yermakov I. P. Ion exchange properties of the root cell walls isolated from the halophyte plants (*Suaeda altissima* L.) grown under conditions of different salinity. Plant Soil 277, 163–174 (2005).

[b33] Liu Y. & Xu R. K. The forms and distribution of aluminum adsorbed onto maize and soybean roots. J. Soils Sediments 15, 491–502 (2015).

[b34] Jones D. L. Organic acids in the rhizosphere-a critical review. Plant Soil 205, 25–44 (1998).

[b35] Vlamis J. & Williams D. E. Ion competition in manganese uptake by barley plants. Plant Physiol. 37, 650–655 (1962). 16655709 10.1104/pp.37.5.650 PMC549850

[b36] Heim A., Luster J., Brunner I., Frey B. & Frossard E. Effects of aluminium treatment on Norway spruce roots: Aluminium binding forms, element distribution, and release of organic substances. Plant Soil 216, 103–116 (2000).

[b37] Zhong K., Xu R. K., Zhao A. Z., Jiang J. & Li H. Adsorption and desorption of Cu(II) and Cd(II) in the tropical soils during pedogenesis in the basalt from Hainan, China. Carbonate. Evaporite. 25, 27–34 (2010).

[b38] McBride M., Sauve S. & Hendershot W. Solubility control of Cu, Zn, Cd and Pb in contaminated soils. Eur. J. Soil Sci. 48, 337–346 (1997).

[b39] Tong X. J., Li J. Y., Yuan J. H. & Xu R. K. Adsorption of Cu(II) by biochars generated from crop straws. Chem. Eng. J. 172, 828–834 (2011).

[b40] Soudek P. . Effect of heavy metals on inhibition of root elongation in 23 cultivars of flax (*Linum usitatissimum* L.). Arch. Environ. Contam. Toxicol. 59, 194–203 (2010). 20174789 10.1007/s00244-010-9480-y

[b41] Wen Y. K. & Shao J. Ionic polarization and metal ion hydrolysis regularity. Chin. Sci. Bull. 22, 267–268 (1977).

[b42] Hardiman R. T. & Jacoby B. Absorption and translocation of Cd in bush beans (*Phaseolus Vulgaris*). Physiol. Plant. 61, 670–674 (2006).

[b43] Martell A. E. & Smith R. M. Critical stability constants (Plenum Press, 1989).

[b44] Bastug A. S., Gokturk S. & Sismanoglu T. 1:1 Binary complexes of citric acid with some metal ions: Stability and thermodynamic parameters. Rev. Inorg. Chem. 27, 53–65 (2007).

[b45] Wang J., Evangelou B. P., Nielsen M. T. & Qagner G. J. Computer-simulated evaluation of possible mechanisms for quenching heavy metal ion activity in plant vacuoles: I. Cadmium. Plant Physiol. 97, 1154–1160 (1991). 16668502 10.1104/pp.97.3.1154 PMC1081135

[b46] Stevens C. J., Dise N. B. & Gowing D. J. Regional trends in soil acidification and exchangeable metal concentrations in relation to acid deposition rates. Environ. Pollut. 157, 313–319 (2009). 18674853 10.1016/j.envpol.2008.06.033

[b47] Li Z. Y., Liu Y., Zheng Y. Y. & Xu R. K. Zeta potential at the root surfaces of rice characterized by streaming potential measurements. Plant Soil 386, 237–250 (2015).

